# Synthesis of Spongy-Like Mesoporous Hydroxyapatite from Raw Waste Eggshells for Enhanced Dissolution of Ibuprofen Loaded via Supercritical CO_2_

**DOI:** 10.3390/ijms16047960

**Published:** 2015-04-09

**Authors:** Abdul-Rauf Ibrahim, Xiangyun Li, Yulan Zhou, Yan Huang, Wenwen Chen, Hongtao Wang, Jun Li

**Affiliations:** Department of Chemical and Biochemical Engineering, College of Chemistry and Chemical Engineering, National Engineering Laboratory for Green Chemical Productions of Alcohols, Ethers and Esters, Xiamen University, Xiamen 361005, China; E-Mails: ghrauf@gmail.com (A.-R.I.); xiangyun068@gmail.com (X.L.); iamrhea14@gmail.com (Y.Z.); yanh1236@gmail.com (Y.H.); wenlimy@163.com (W.C.); wanght@xmu.edu.cn (H.W.)

**Keywords:** hydroxyapatite, waste eggshell, ibuprofen, drug delivery, supercritical loading

## Abstract

The use of cheaper and recyclable biomaterials (like eggshells) to synthesize high purity hydroxyapatite (HAp) with better properties (small particle size, large surface area and pore volume) for applications (in environmental remediation, bone augmentation and replacement, and drug delivery systems) is vital since high-purity synthetic calcium sources are expensive. In this work, pure and mesoporous HAp nanopowder with large pore volume (1.4 cm^3^/g) and surface area (284.1 m^2^/g) was produced from raw eggshells at room temperature using a simple two-step procedure. The control of precursor droplets could stabilize the pH value of the reaction solution, because of the size of the needle (of the syringe pump used for precursor additions) leading to production of HAp with high surface area and pore size. The as-produced HAp revealed high ibuprofen (as a model drug) loading (1.38 g/g HAp), enhanced dissolution and controllable release of the drug via solute-saturated supercritical carbon dioxide.

## 1. Introduction

As a result of potential applications in several fields (such as electronics, information technology, ceramics, medicine, pharmacology, tissue engineering as well as environmental control), substantial research has been advanced towards the development of inorganic nanocrystals [1]. Concerns about environmental issues and harvesting of bones for surgery have been on the center stage of global discussions as such development of environmentally friendly techniques and humane processes to meet best practice requirements of these areas has attracted widespread attention. Accordingly, production and application of several inorganic materials in view of meeting these standards have been promoted [2]. Hydroxyapatite (HAp; Ca_5_(PO_4_)_3_OH), one of such important inorganic materials, has been of interest owing to its excellent biocompatibility, affinity for biopolymers and high osteogenic potential [3,4]. In fact, HAp has been used in numerous fields. For instance, it has been used for the removal of heavy metals in drinking and waste waters, for the production of bioceramics and powder for tissue engineering, dental, drug delivery and other medical applications [1,5,6]. However, high-purity synthetic calcium sources are somewhat expensive hence the use of HAp produced from these sources (synthetic calcium) in environmental remediation, and bone augmentation and replacement (in surgery) is expensive [7]. Besides, it has been shown that resorption of HAp derived from synthetic calcium is quite different from that of the bone mineral [8]. Therefore, alternative procedures to produce HAp from cheap and recyclable biological materials have been pursued [9,10,11]. These are expected to attract more attention in the near future because of the better physicochemical properties of HAp generated from biogenic sources [3]. The use of waste eggshells has been dominant [12] among the alternative procedures. Indeed, using waste eggshells to produce HAp offers several advantages: (1) it reduces the cost of production [9,10,11]; (2) it offers HAp that is very compatible with the human bone and hard tissues [8]; and (3) it is an environmental and waste control measure [2].

The eggshell constitutes about 10%–11% of the overall weight of an egg; this is a huge “bio-calcium” source [13]. For example, China is the world’s largest producer of eggs with nearly 45% of the global egg production capacity [14]. Traditionally, the eggshells are dumped or incinerated after consumption of the egg contents. This is a big environmental issue; however, it is also an immense biological calcium (bio-calcium) source for countless applications. One of such applications (as stated) is conversion of waste eggshells into HAp. Nonetheless, previous procedures for converting eggshells into HAp have generated impure products with larger particle sizes, low surface areas and very low pore volumes [15]. Consequently, the ratio of surface to volume in such HAp is very low leading to negligible surface free energy [13]. This makes production of HAp nanopowder (HApNP) from eggshells with higher surface areas and smaller particle sizes without templates difficult although there were some published papers related to the fabrication of HApNP using templates [16]. Yet, these parameters are vital as they determine the characteristics of the HAp and its final applications. In this regard, specific properties such as higher surface area, pore volume, small particle sizes and biocompatibility are much desired.

We produced HApNP with a high surface area (as large as 212.4 m^2^/g) at room temperature (RT) from waste eggshells which was dissolved into Ca(NO_3_)_2_ to react with dilute phosphoric solution [17]. We also produced rod-like HAp with high surface area (as large as 160.1 m^2^/g) and large pore volume (as large as 1.0 cm^3^/g) at RT from waste eggshells which was calcined into CaO to react with dilute phosphoric solution [18] with the aid of a syringe pump to stabilize the reaction pH. Both HAp materials showed fast and very high maximum adsorption to Pb^2+^ [18]. Surely, without the use of templates or structure directing agents, HAp with low BET surface areas (31–52 m^2^/g) is normally produced from waste eggshells by typical hydrothermal and sol-gel procedures [19]. This situation also gave birth to renewed and tremendous efforts to produce HAp with better surface properties for various applications. For instance, HApNP with 127 m^2^/g surface area was prepared from calcium d-gluconate with assistance of a polymer stabilizer [20]. HAp powder with 130 m^2^/g surface area and 40 nm mean particle size was produced by a reverse micelle templating procedure [21]. HApNP of a hollow core and mesoporous shell structure and an oval shape with surface area of 163.2 m^2^/g and pore volume of 0.53 cm^3^/g by using a colloidal templating method was produced [22]. In this work, we combined the previous studies [17,18] with the deliberate control of the pH of the reaction solution (Ca(NO_3_)_2_ from eggshells and dilute phosphoric solution) via NH_4_OH to explore further improvement of the HAp properties. Several phosphate solutions (H_3_PO_4_, Na_2_HPO_4_, NaH_2_PO_4_ and NH_4_PO_4_) were examined.

Applications of HAp are numerous and varied as mentioned. In this study, we applied the as-synthesized HAp as a drug carrier for drug delivery systems (DDS). Specifically, we investigated the loading of a model drug (ibuprofen) into the as-produced HAp via the solute-saturated supercritical carbon dioxide approach [23] and its *in vitro* release. A recent study showed that a HAp/MCM-48 composite could evidently increase the release rate of ibuprofen compared to MCM-48 [24]. In another study, ibuprofen was dispersed in mesoporous silica using supercritical CO_2_ as the solvent and showed a maximum drug loading of 42.0% (g ibuprofen/g silica) at 17 MPa and 37 °C [25].

## 2. Results and Discussion

### 2.1. X-ray Diffraction (XRD) Analysis

**Figure 1:**
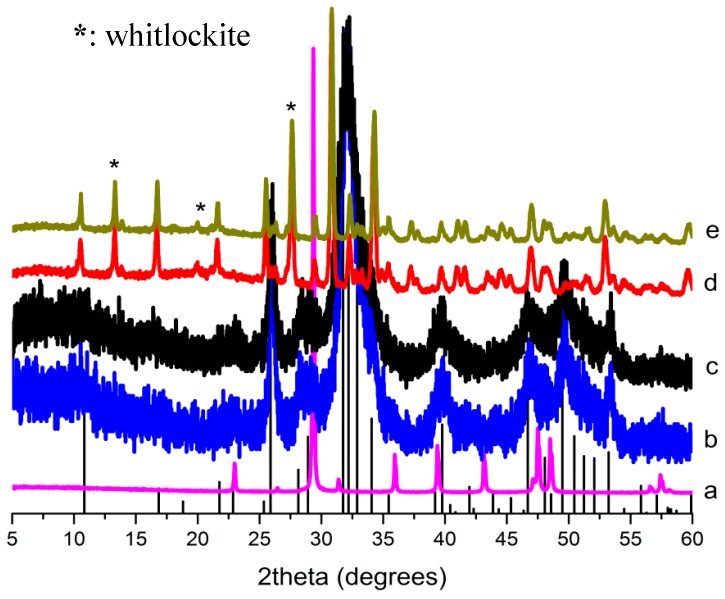
XRD Patterns of (a) calcite from waste eggshell; hydroxyapatite (Hap) produced using (b) Na_2_HPO_4_ and (c) H_3_PO_4_ (HAp284PA); and the calcined HAp284PA at (d) 700 °C and (e) 950 °C; bars: HAp reference (ICSD-PDF2: 01-084-1998).

Figure 1 shows the XRD patterns for the raw eggshells (Figure 1a), a HAp sample from H_3_PO_4_ (HAp284PA, which has a specific surface area of 284.1 m^2^/g) (Figure 1b), another HAp sample from Na_2_HPO_4_ (Figure 1c) and the corresponding calcined samples (Figure 1d,e). Note that the patterns for the samples from the other phosphate precursors were very similar to these patterns hence not shown. The pattern for the raw eggshells revealed respective calcite peaks corresponding to ICSD-PDF2 card No. 01-083-0578. Interestingly, the patterns for the products showed no precursor peaks; all the peaks were indexed to HAp (ICSD-PDF2: 01-084-1998) with strong diffractions at about 31.773° in the 2 1 1 plane [26]. Analysis of the patterns for the heat treated samples revealed that they were stable albeit little transformation; for instance, the samples produced from phosphoric acid and calcined at 700 °C and 950 °C for 2 h are shown in Figure 1d,e respectively. XRD analysis showed little transformation of the pure HAp phase into another calcium phosphate phase (whitlockite; it is tricalcium phosphate, a common bone substitute [27]). However, the quantity of this new phase (whitlockite) was trivial (about 4.5% at 700 °C and 5.2% at 950 °C). Yet, the crystallinity of the samples increased after the calcination.

### 2.2. Scanning Electron Microscopy (SEM) Analysis

**Figure 2:**
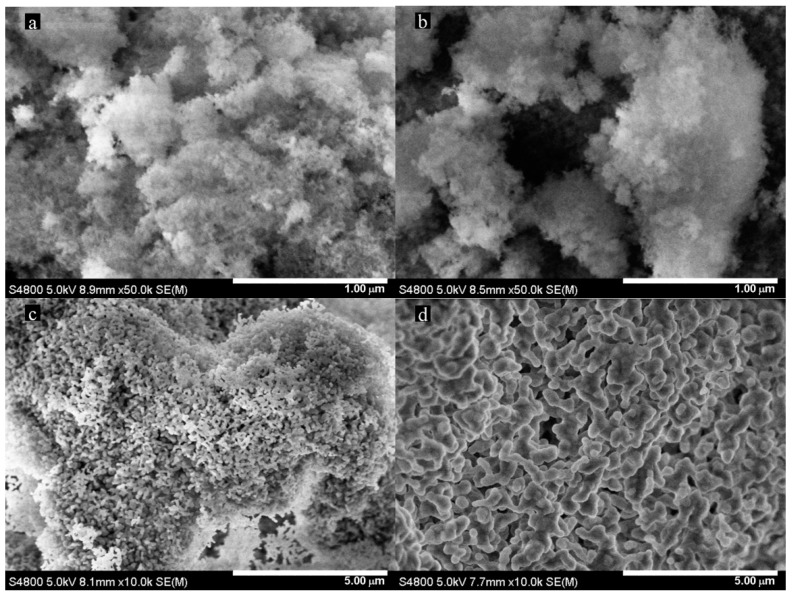
SEM images of HAp produced using (**a**) Na_2_HPO_4_ and (**b**) H_3_PO_4_ (HAp284PA); and the calcined HAp284PA at (**c**) 700 °C and (**d**) 950 °C.

SEM images of the HAp sample from Na_2_HPO_4_ and the HAp284PA sample and the calcined products are shown in Figure 2. Again, the patterns for the other phosphate precursors were similar to that from H_3_PO_4_.

The images revealed production of spongy-like morphology, particularly for the phosphoric acid sample (Figure 2b). However, this morphology was lost in the phosphoric acid sample after the calcination leading to porous particles at 700 °C (Figure 2c) and thick porous particles at 950 °C (Figure 2d). Porous biogenic HAp ceramics are known to facilitate and enhance circulation of body fluid when applied on metal coatings for implants and in surgery [7].

### 2.3. Functional Group Analysis

Fourier transform infrared spectroscopy (FTIR) spectrum (not shown) showed all the various vibrational bands [28] of the HAp284PA sample. For this case, a resolution of 4 cm^−1^ from 400 to 4000 cm^−1^ was instituted using KBr method. Note that the spectra for the samples from all the phosphate precursors were similar and contained carbonate bands indicating the substitution of carbonate ions at the phosphate sites [29]. Note that the FTIR spectrum is similar to the previously [17,18] reported ones.

### 2.4. Brunner-Emmet-Teller (BET) Results

The specific surface area, pore size and pore volume from BET and the crystallite size from XRD for the samples are listed in Table 1.

**Table 1:** Results of BET analysis of the produced HAP powders.

Sample	Surface Area (m^2^/g)	Pore Volume (cm^3^/g)	Pore Size (nm)	ACS ^a^ (nm)
Na_2_HPO_4_	205.0	0.71	12.4	9
NaH_2_PO_4_	217.8	0.96	15.2	8
(NH_4_)_3_PO_4_	226.9	0.85	13.0	7
H_3_PO_4_(HAp284PA)	284.1	1.4	17.2	7
Calcined at 700 °C	165.1	0.83	18.7	12
Calcined at 950 °C	57.1	0.47	20.2	46

^a^ Average crystallite size (ACS) was obtained using Jade 6.0 software based on XRD patterns.

All the samples were mesoporous and had large specific surface areas and pore volumes which may be attributed to their spongy-like morphology. However, the HAp284PA sample obtained using H_3_PO_4_ exhibited the highest surface area and pore parameters with 284.1 m^2^/g surface area, 17.2 nm pore size and 1.4 cm^3^/g pore volume. These are the highest values so far reported in the literature for HAp produced from waste eggshells. We reported the synthesis of HAp with 212.4 m^2^/g surface area, 16.8 nm pore size and 0.98 cm^3^/g pore volume previously [17]. Note that similar reaction conditions and precursors were employed. Yet the difference in the surface parameters (Figure 2b) suggests synthesis of much finer and interconnected fibers currently than previously reported [17] perhaps due to the different chemicals used for the pH controls. Recall that NaOH pallets were used for the previous case whereas NH_4_OH solution was used currently. To the best of our knowledge, the results we obtained are even better than some values reported for the production of HApNP by the use of synthetic and aqueous calcium sources using block polymers, templates, emulsions and structure directing agents [29]. For instance, in spite of prolonged and complicated steps, Shum *et al.* synthesized mesoporous hydroxyapatite powder by double emulsion droplets technique using synthetic calcium nitrate tetrahydrate and phosphoric acid, and reported a BET surface area of 162.8 m^2^/g [30]. Furthermore, Hoffmann *et al.* precipitated HAp by a biomimetic (enzyme catalytic) method using alkaline phosphatase, CaCl_2_ and MgCl_2_, and reported a surface area of 173 m^2^/g [31]. Meanwhile, the crystallite sizes (from the XRD analysis) confirmed the very small (7–9 nm) nature of the particles produced (Table 1). Additionally, the nitrogen adsorption and desorption isotherms (not shown) for the various powders exhibited mesoporous type IV isotherms with hysteresis loops.

HAp powder with high surface area possesses enhanced sinterability kinetics leading to enhanced mechanical properties [1]. For nanometric particle sizes (<20 nm) and higher surface areas, microemulsion and other complex techniques have been used [32]. HAp powders with smaller particles sizes (<10 nm) and higher surface areas have greater number of atoms (>50%) on the surface leading to larger surface to volume ratio. This is particularly important for densification, drug delivery and bone morphogenesis [33]. The dimensions of biological apatite in calcified tissues are always in the range of a few to hundreds of nanometers, with the smallest building blocks on the nanometer size scale; this is a natural selection since nanostructured materials provide the capability for specific interactions with proteins [34]. Notwithstanding, previous procedures instituted to obtain these superstructures were not only time consuming (leading to high operational costs) but they usually employed toxic templates and solvents [35]. Synthesis of the spongy-like HApNP by this simple procedure may offer biosorbents with effective sorption abilities because of the higher surface area [36], bioceramics with excellent reinforcement due to enhanced mechanical properties [37], and higher drug loading when applied as drug carriers [38].

### 2.5. Effect of Needle Size

The experiments reported in Table 1 were implemented with a 1.2 × 38 mm (OD × length) needle. Therefore, we explored the effect of needle size on the properties of the samples produced (Table 2) from phosphoric acid. As can be seen, the surface area appears to be dependent on the size of needle. It is clear that the size of needle controls the phosphoric acid or phosphate solution drop rate and droplet size; therefore, an appropriate needle may stabilize the pH of reaction solution. The surface area increased from 154.1 m^2^/g with the 0.45 × 16 mm needle (pH of the reaction solution varied from 9.79 to 10.47) to a maximum of 284.1 m^2^/g with the 1.2 × 38 mm needle (pH of the reaction solution stabilized within 10.00 to 10.47) and then begun to decrease to 170.9 m^2^/g with the 41 × 113 mm needle (pH of the reaction solution varied from 9.95 to 10.47). The pore volume seemed to follow the same trend however, the pore size showed no clear cut dependence on the needle size. Using the 1.2 × 38 mm needle, the average particle size (from the BET analysis) exhibited the smallest value (6.7 nm), while those from other needles were between 10 and 12 nm. Yet, the average crystallite size (from the XRD patterns) from the 1.2 × 38 mm needle was 7 nm, whereas that of the other needles was 9 nm. Clearly, the size of needle seemed to have positive effect on the surface parameters but the length was less significant. For instance, needles with the same size but different lengths (0.7 × 32 and 0.7 × 80 mm needles) did not show substantial difference in surface areas (166.1 and 166.8 m^2^/g respectively). Similarly, the length did not have obvious impact on the crystallite sizes (7 nm for both 0.7 × 32 and 0.7 × 80 mm needles). However, needles with similar lengths but different sizes (0.9 × 38 and 1.2 × 38 mm) revealed considerable variation in the surface areas (170.8 and 284.1 m^2^/g respectively), even with just 0.1 mm difference in needle size (0.7 × 32 mm yielded 166.1 m^2^/g and 0.8 × 32 mm gave 169.3 m^2^/g). Likewise, it affected the crystallite size: the 0.9 × 38 mm needle yielded an average crystallite size of 9 nm whereas the 1.2 × 38 mm needle produced particles with an average crystallite size of 7 nm (see Table 2).

**Table 2:** Effect of needle size on BET surface area, pore size and crystallite size.

Needle Size (mm)	*ND* ^a^	Lowest pH ^b^	Surface Area (m^2^/g)	Pore Volume (cm^3^/g)	Pore Size (nm)	ACS (nm)
0.45 × 16	na ^c^	9.79	154.1	0.76	19.5	7
0.6 × 25	na	9.83	162.1	0.78	18.0	8
0.7 × 32	180	9.92	166.1	0.88	19.4	7
0.7 × 80	150	9.92	166.8	0.93	20.0	7
0.8 × 32	139	9.93	169.3	0.75	16.2	8
0.9 × 38	120	9.94	170.8	0.78	16.8	9
1.2 × 38	98	10.00	284.1	1.39	17.2	7
20 × 125	46	9.97	189.4	0.61	12.1	8
41 × 113	44	9.95	170.9	0.83	17.9	7

^a^ *ND* is the number of drops counted within a minute (drop/min) at 3.33 mL/min (50 mL of phosphoric acid solution added at 200 mL/h into 50 mL of Ca (NO_3_)_2_ solution); ^b^ the lowest pH values attained for the various reaction solutions (the highest pH value before addition of the phosphoric acid solution was 10.47); ^c^ na: drops were so fast that it was not possible to count.

### 2.6. Thermal Stability

Thermal stability and weight loss of the HAp284PA sample were studied by thermogravimetric analysis (TGA). The total weight loss was about 11.8%. The product degraded slowly from RT to about 200 °C resulting in a weight loss of about 7.3% attributed to loss of water. Further heating to 500 °C caused a weight loss of 3.1% while weight loss of about 1.4% was realized at 800 °C. However, there was no discernible weight loss above this temperature indicating phase stability due to completion of decomposition and perhaps, liberation of bonded liquids and gases. Note that the weight loss data is similar to the previously reported data [17].

### 2.7. Loading Composition

**Figure 3:**
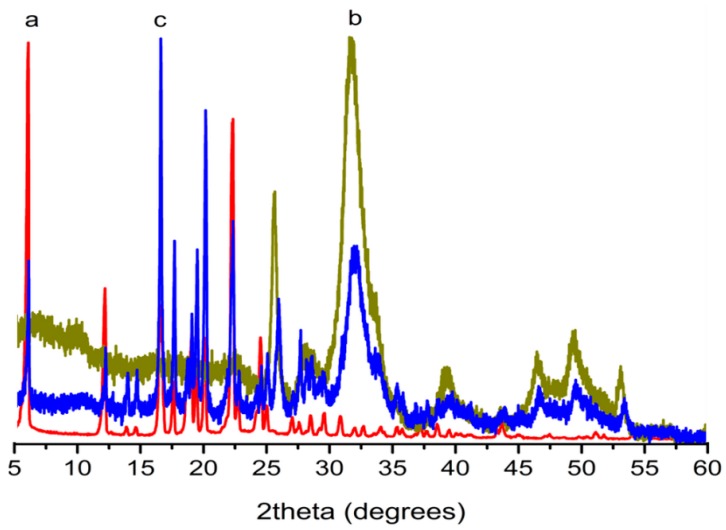
XRD patterns for (a) pure ibuprofen; (b) as-HAP (HAp284PA); and (c) ibuprofen-HAp mixture samples.

The weight composition and phase purity of the ibuprofen loaded sample (loaded at 18.0 MPa in HAp284PA) were studied by XRD and compared with the pure ibuprofen (Figure 3). As can be seen, the sample contained only ibuprofen and HAp peaks indicating its purity. The ibuprofen was indexed to ICCD PDF2: 00-032-1723 with a monoclinic crystal system and P21/c space group (a = 14.667, b = 7.8990, c = 10.731).

From the UV analysis (see Section 3.5), the mass fraction of ibuprofen in the mixture (loaded at 18.0 MPa in HAp284PA) was determined to be 1.38 ± 0.15 g/g HAp which is much larger than the loading in silica reported in [25] due to the solute-saturated supercritical fluid loading process and the HAp material used here.

**Figure 4:**
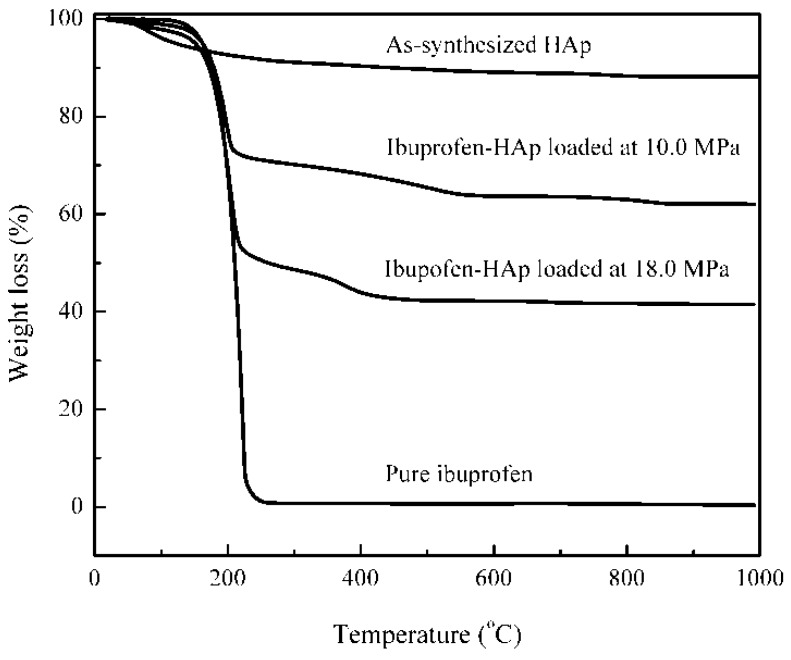
TG curves for the as-produced HAp, pure ibuprofen and ibuprofen loaded samples.

From the TG curve of pure ibuprofen (Figure 4), all ibuprofen (100%) was burnt out by 250 °C. However, the pure HAp material was stabilized after 800 °C but showed a weight loss of about 3.4% (from 250 to 800 °C). For the ibuprofen-HAp (HAp284PA) sample loaded at high pressure (18.0 MPa), the weight loss from 250 to 800 °C (8.3%) was attributed to loss of the HAp material and the ibuprofen inside (loaded into) the pores of the HAp since all the ibuprofen would be lost by 250 °C. Thus, the amount of ibuprofen on the surface of the HAp particles (adsorbed) may be obtained from the weight loss from RT to 250 °C and from 250 to 800 °C as:

(1) $$l_{\text{Ibu,s}}=\frac{{\left[W_{\text{HAp-Ibu}}-W_{\text{HAp}}\right]}_{\text{RT-250}}}{m_{\text{HAp}}}$$

(2) $$l_{\text{Ibu,i}}=\frac{{\left[W_{\text{HAp-Ibu}}-W_{\text{HAp}}\right]}_{\text{250-800}}}{m_{\text{HAp}}}$$

where *l*_Ibu,s_ represents the ibuprofen loaded on the surface of the HAp (g ibuprofen/g HAp) and *l*_Ibu,__i_ denotes the ibuprofen loaded into the pores of the HAp (g ibuprofen/g HAp); *m*_HAp_ represents the mass of HAp (g); *W* denotes the weight loss (g) obtained from the TG curves; *W*_HAp-Ibu_ is the weight loss of the HAp-ibuprofen sample from RT to 250 °C or from 250 to 800 °C and *W*_HAp_ is the weight loss of HAp from RT to 250 °C or from 250 to 800 °C.

In the case of the higher pressure loading at 18.0 MPa, the TG curve provided that *W*_HAp_ = 8.3% × *m*_T_ (*m*_T_ is the weight of the ibuprofen-HAp sample) and *W*_HAp-Ibu_ = 49.4% × *m*_T_ from RT to 250 °C; the curve also provided that *W*_Hap_ = 3.4% × *m*_T_ and *W*_HAp-Ibu_ = 9.0% × *m*_T_ from 250 to 800 °C. The final weight loss of the ibuprofen-HAp sample is 58.5% from the TG curve; therefore, *m*_HAp_ = 41.5% × *m*_T_. Accordingly, *l*_Ibu,s_ = 0.99 g ibuprofen/g HAp and *l*_Ibu,i_ = 0.16 g ibuprofen/g HAp. Note that over 86% of the total ibuprofen loaded was located on the surface of the HAp particles but that the loading may be both adsorption and agglomeration. Clearly, the amount of ibuprofen loaded from the TG analysis (0.99 + 0.16 = 1.15 g ibuprofen/g HAp) is approximately in agreement with the amount of ibuprofen from the UV analysis (1.38 g ibuprofen/g HAp).

For the lower pressure loading (10.0 MPa, with the same conditions), *l*_Ibu,s_ = 0.50 g ibuprofen/g HAp and *l*_Ibu,i_ = 0.16 g ibuprofen/g HAp were obtained (76% of the total ibuprofen loaded was located on the surface of the HAp particles). The TG results from the two loadings indicated that the loading on the surface (0.99 g/g for 18.0 MPa against 0.50 g/g for 10.0 MPa) was more sensitive to the pressure than the loading into the pore (0.16 g/g for both). This was due to the fact that the loading on the surface was not exclusively adsorption; there could have been impregnation or/and agglomeration. These later mechanisms (impregnation or/and agglomeration) would arise from the precipitation of ibuprofen dissolved in supercritical CO_2_ (sensitive to the pressure).

### 2.8. Dissolution Test

**Figure 5:**
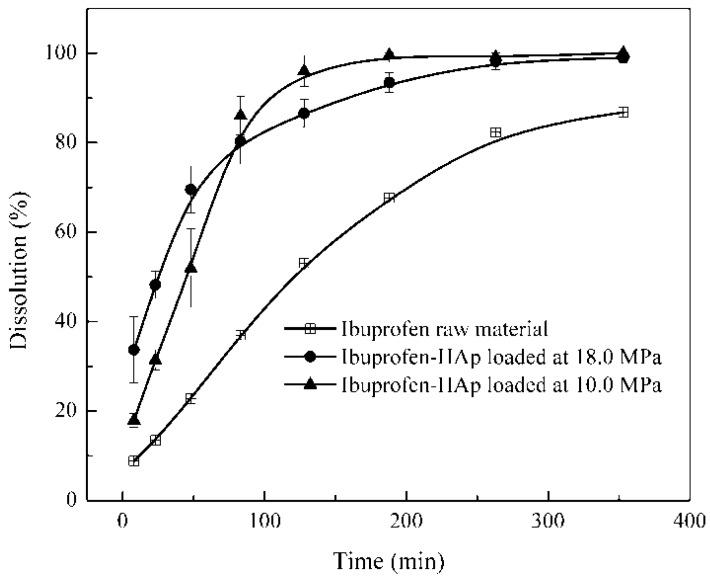
Dissolution profiles for ibuprofen raw material and ibuprofen-HAp samples in a simulated gastric fluid.

The dissolution profiles of the tested samples are shown in Figure 5. About 9%, 20% and 37% of ibuprofen were released from the raw material with the lower pressure (10.0 MPa) and the higher pressure (18.0 MPa) loaded samples respectively, within the first 10 min of dissolution. The relatively low dissolution of the ibuprofen raw material may be due to its relatively large particle sizes and low solubility in the solution with pH < 7 [39]. Within 50 min, the dissolution of the higher pressure loaded sample (≈70%) was obviously faster than those of the lower pressure loaded sample (≈57%) and the ibuprofen raw material (≈24%). These values (57% and 70%) suggest that the release of ibuprofen was mainly from the surface of the HAp particles (about 75% and 86% of the total ibuprofen on the HAp surface for the lower pressure loaded sample and higher pressure loaded sample respectively). Subsequently, the dissolution of ibuprofen from the pores of the higher pressure loading sample slowed down although that from the lower pressure loading sample was still faster. Interestingly, the dissolution from both pressure loadings was the same (78%) in about 80 min but that of the raw material was still low (35%). The phenomena suggest that the higher pressure loading would deposit ibuprofen deep into the pores of HAp which may be more difficult to release (slow release). Obviously, the dissolution from both pressure loadings was fast (with complete release of ibuprofen in about 350 min). They were even faster than the dissolution (91.7% in 353 min) of ibuprofen nanoparticles (260 nm) from a previous supercritical fluid-based method [39]. These results therefore confirm the control release and evidently, enhanced dissolution of ibuprofen because the supercritical CO_2_ loading could easily alter the amount of ibuprofen on the surface/in the pores of the HAp particles.

## 3. Experimental Section

### 3.1. Materials

Waste eggshells were collected from the Furong canteen in Xiamen University. They were pretreated as before [17] (washed with detergent, hexane and distilled water, and finally dried at 80.0 °C overnight). Phosphoric acid (purity ≥ 85%), disodium hydrogen phosphate dodecahydrate (≥99.0%), sodium dihydrogen phosphate dehydrate (≥99.0%), ammonium phosphate tribasic trihydrate (≥95.0%) and nitric acid (68%) were purchased from Sinopharm Reagent Co., Ltd., Shanghai, China. Ammonia solution (85%) was purchased from Guangdong Guanghua Sci-Tech Co., Ltd., Guangzhou, China.

### 3.2. Synthesis of HApNP

The synthesis of the HApNP was achieved using a two-step procedure (as stated earlier). In the first step, appropriate weight of pretreated eggshells were dissolved in concentrated nitric acid at RT under a high speed mechanical stirrer (IKA RW, 20 DS 25; 800–1000 rpm) to produce the Ca(NO_3_)_2_:

(3) $${\text{CaCO}}_{\text{3(eggshells)}}+{\text{2HNO}}_{\text{3(aq)}}\to {\text{Ca}({\text{NO}}_{3})}_{\text{2(sol)}}+{\text{H}}_{\text{2}}{\text{O}}_{\left(l\right)}+{\text{CO}}_{\text{2(g)}}↑$$

In the second step, 50 mL of 0.06 M dilute phosphate solution (from H_3_PO_4_, Na_2_HPO_4_, NaH_2_PO_4_ or (NH_4_)_3_PO_4_) was prepared and added at the rate of 200 mL/h using a syringe pump (TCI-IV, Guanxi, China) at RT under vigorous agitation (450–500 rmp) to react with 50 mL of 0.1 M calcium nitrate solution Ca(NO_3_)_2_:

(4) $${\text{5Ca}}^{\text{2+}}+{\text{3PO}}_{\text{4}}^{3-}+{\text{OH}}^{-}\to {\text{Ca}}_{\text{5}}{\left({\text{PO}}_{\text{4}}\right)}_{\text{3}}\text{OH}↓$$

**Figure 6:**
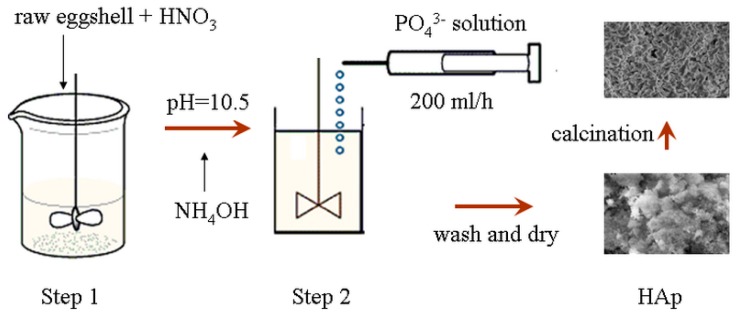
Illustration for the preparation of HAp.

The pH of the reaction solution was adjusted to about 10.5 before commencement of each reaction using NH_4_OH solution. The products were finally washed repeatedly with distilled water due to the presence of water soluble by-products and then with ethanol to remove traces of water, vacuum dried (80 °C, 12 h) and sintered (at 700 and 950 °C) in an oven (Jing Hong, Shanghai, China) for 2 h. Figure 6 shows the illustration of the preparation procedure.

The difference between this procedure and similar processes in the literature is in the use of (1) high speed mechanical stirrer to facilitate froth control in the first step; and (2) syringe pump to facilitate pH control during the second step. Dissolution of raw eggshells into concentrated nitric acid is usually characterized by formation of froth [40] which is difficult to handle. Nevertheless, the eggshells were added intermittently over a period of 2 h. On the other hand, the syringe pump offered control over addition of the phosphorous precursors with precision and consistency instead of the usual drop-wise addition.

### 3.3. Characterization of HApNP

Specific surface area, pore volume and pore size of the powders were determined by BET (Micromeritics ASAP 2020, Norcross, GA, USA), and the nitrogen adsorption and desorption isotherms were obtained by performing isothermal physical sorption at 77.3 K. Thermal stability analysis was studied by thermogravimetric analysis-differential thermal analysis TG-DTA (SDTQ600, TA Instruments, New Castle, DE, USA) from RT to 1000 °C using a heating rate of 10 °C/min. Morphology of the particles was studied with SEM (Hitachi, S-4800, Tokyo, Japan). Phase purity was analyzed using XRD (Rigaku Ultima IV, Tokyo, Japan) using a step size of 0.02° in 10 s from 5°–60° in a reflection mode with Cu K_α_ radiation. Crystallite structure was refined with X’pert Highscore Plus software using Rietveld refinement and the mean crystallite sizes were calculated from Jade 6.0 software using XRD data (based on the 2 1 1 plane). Various functional groups of the samples were determined using FT-IR (Nicolet 330, Thermo Electron Corporation, Waltham, MA, USA) with a resolution of 4 cm^−1^ over a range of 400–4000 cm^−1^ with the KBr pellet method.

### 3.4. Loading of Ibuprofen

**Figure 7:**
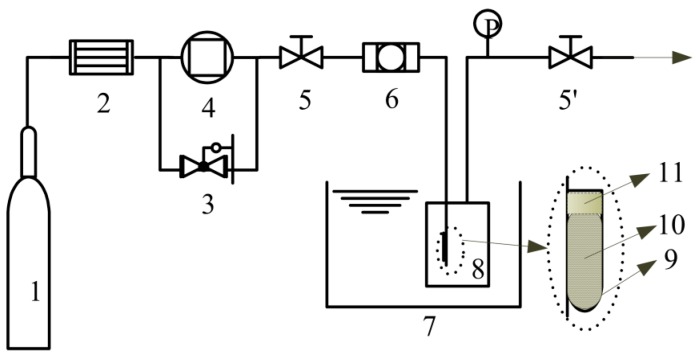
Schematic diagram of the solute-saturated supercritical loading process; (1) CO_2_ cylinder; (2) dryer; (3) back pressure valve; (4) compressor; (5,5’) valves; (6) filter; (7) water bath; (8) loading vessel; (9) tube ; (10) ibuprofen loaded; (11) cotton; (P) pressure gauge.

We conducted a higher pressure (18.0 MPa) and a lower pressure (10.0 MPa) loading of ibuprofen into the as-prepared HAp284PA sample (see Table 1) at 40.0 °C in 24 h. The experimental apparatus is shown in Figure 7.

About 12.0 g of ibuprofen was loaded into the high-pressure vessel (110 mL) such that the ibuprofen was enough to saturate with supercritical CO_2_; this gives a solute-saturated supercritical fluid loading process [23]. In the meantime, about 30 mg HAp was placed into a plastic centrifuge tube with holes on the cover to facilitate passage of the CO_2_ and adequate amount of cotton was then placed on top of the HAp in the tube to prevent the powder from escaping. The loading vessel was put in a water bath (±0.1 °C) and heated till thermal equilibrium at 40.0 °C. Supercritical CO_2_ was introduced into the loading vessel with controlled pressure by a self-made back pressure valve (±0.1 MPa) to dissolve the ibuprofen and transport it to the HAp through the cotton. The contact time was set to 24 h during which absorption-desorption equilibrium was attained after which the supercritical CO_2_ was slowly released (about 0.1–0.5 MPa/min).

### 3.5. Composition Analysis

In order to calculate the mass fraction of ibuprofen loaded into the HAp284PA sample, a standard curve was instituted with the aid of a UV detector (UV 310) at 220 nm fixed wave length [23]. The standard curve was later used for the dissolution test. For this purpose, exactly 10 mg of the ibuprofen-HAp sample (loaded at 18.0 MPa) was added into 200 mL buffer solution and scanned over the fixed UV wave length to determine the concentration.

TG analysis was also implemented to determine the extent of ibuprofen loaded into the HAp, particularly, to determine the nature of loading involved (adsorption, impregnation or agglomeration) [23]. These mechanisms are important for controlled drug delivery. To determine the location of ibuprofen in the HAp, TG study of the pure ibuprofen and the HAp-ibuprofen mixture samples was conducted as stated earlier (Section 3.3).

### 3.6. Dissolution Test

Dissolution tests were conducted on the pure ibuprofen raw material and the ibuprofen-HAp composites in a simulated gastric fluid (potassium chloride-hydrochloric acid Buffer solution; pH = 1.4 ± 0.02) using a test apparatus (RCA-1A, Huanghai Medicine & Drug Testing Instruments Co., Ltd., Shanghai, China) at 37 ± 0.5 °C with a paddle speed of 100 ± 1 rpm. Exactly 6 mg ibuprofen raw material and about 12 mg of each composite (considering the mass fraction of ibuprofen in the mixtures) were used for the tests. About 8 mL of the solution (after the desired dissolution time) was filtrated through a 0.45 μm membrane filter and analyzed with the UV detector at a fixed wavelength (220 nm) to determine the amount of dissolved ibuprofen (tests were repeated three times).

## 4. Conclusions

A two-step technique for converting waste eggshells as bio-calcium source to synthesize mesoporous HApNP was explored. Based on the results, we conclude that HApNP with a large pore volume (1.4 cm^3^/g) and surface area (284.1 m^2^/g) was produced. We also conclude that these results are the best among values reported for generating HAp from waste eggshells in the literature. About 1.38 g ibuprofen/g HAp was loaded (with over 85%) on the surface of the HAp. The ibuprofen loaded exhibited enhanced (faster) dissolution and controlled release due to the adjustable partition of the ibuprofen on the surface and in the pores of the HAp (caused by the loading technique).
